# Supplementary figures and images for: Novel phenotype of Wolbachia strain wPip in Aedes aegypti challenges assumptions on mechanisms of Wolbachia-mediated dengue virus inhibition
Source: PLoS Pathog. 2020 Jul 29;16(7):e1008410. doi: 10.1371/journal.ppat.1008410 (PMC7416964; doi:10.1371/journal.ppat.1008410)

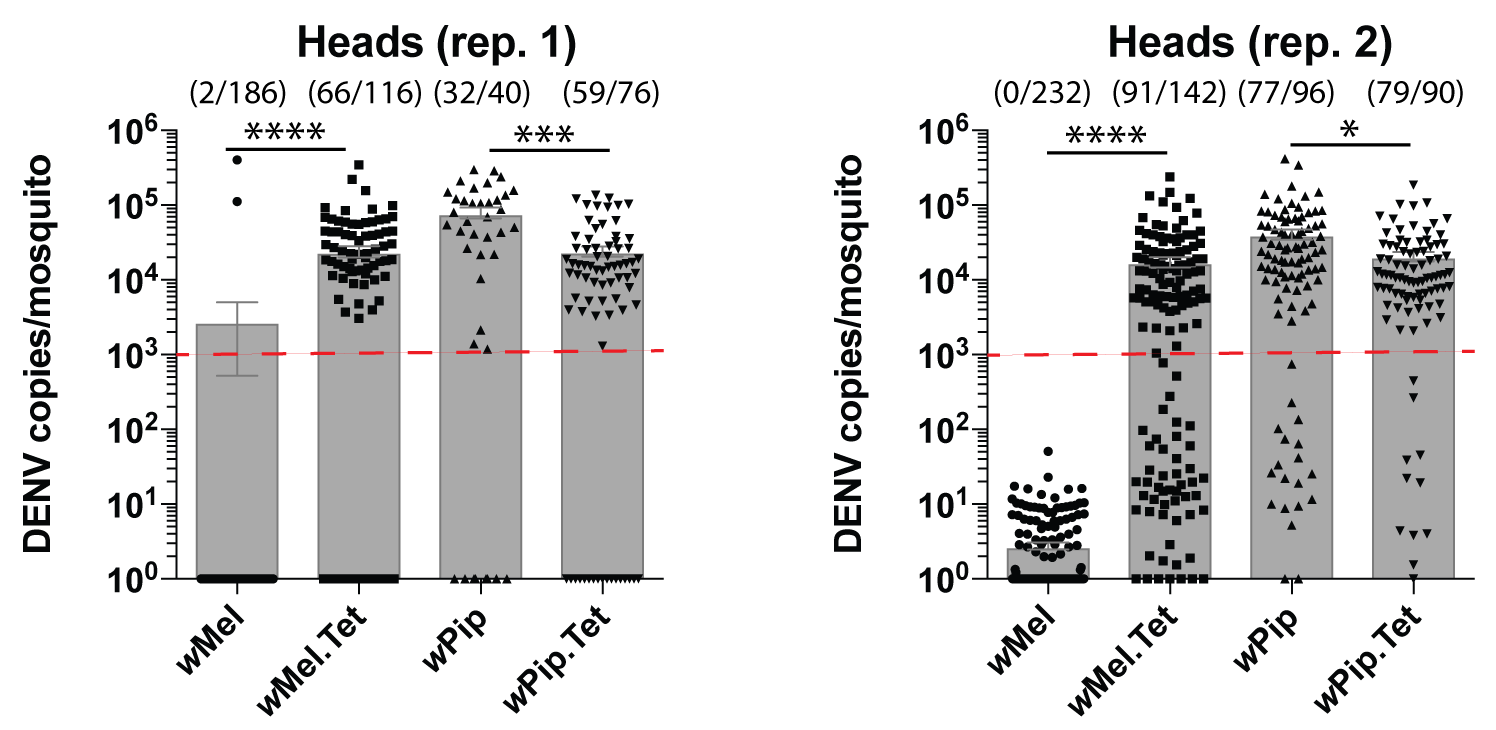

Supplement: S1 Fig — Seven-day old female mosquitoes were fed a blood meal containing DENV-3 (2.09 x 10^7^ TCID50/ml rep. 1 and 3.39 x 10^6^ TCID50/ml rep. 2) and incubated for 14 days. DENV genome copies were determined by qRT-PCR for each head as a measure of viral dissemination. Data are the mean viral genome copies per mosquito head ± SEM, with individual data points overlaid, and were generated from 2 independent experiments (rep. 1 and rep. 2). Statistical analyses were performed using a Mann-Whitney test where * p < 0.05, ***p < 0.001, ****p < 0.0001. Red line indicates LOD95 of the qRT-PCR reaction. Zero values have been plotted as 100 (1) to allow visualization on the log10 scale. Numbers in parentheses above the bars indicates the number of mosquito heads scored positive for DENV per total engorged mosquitoes. Fisher’s exact test indicates significantly more mosquitoes show disseminated virus in the wMel.Tet cohort compared to the wMel cohort (p<0.00001 rep. 1 and rep. 2), but no significant difference in the number of mosquitoes showing disseminated virus between wPip and wPip.Tet cohorts (p>0.05 rep. 1 and rep. 2). (TIF) [file ppat.1008410.s001.tif]

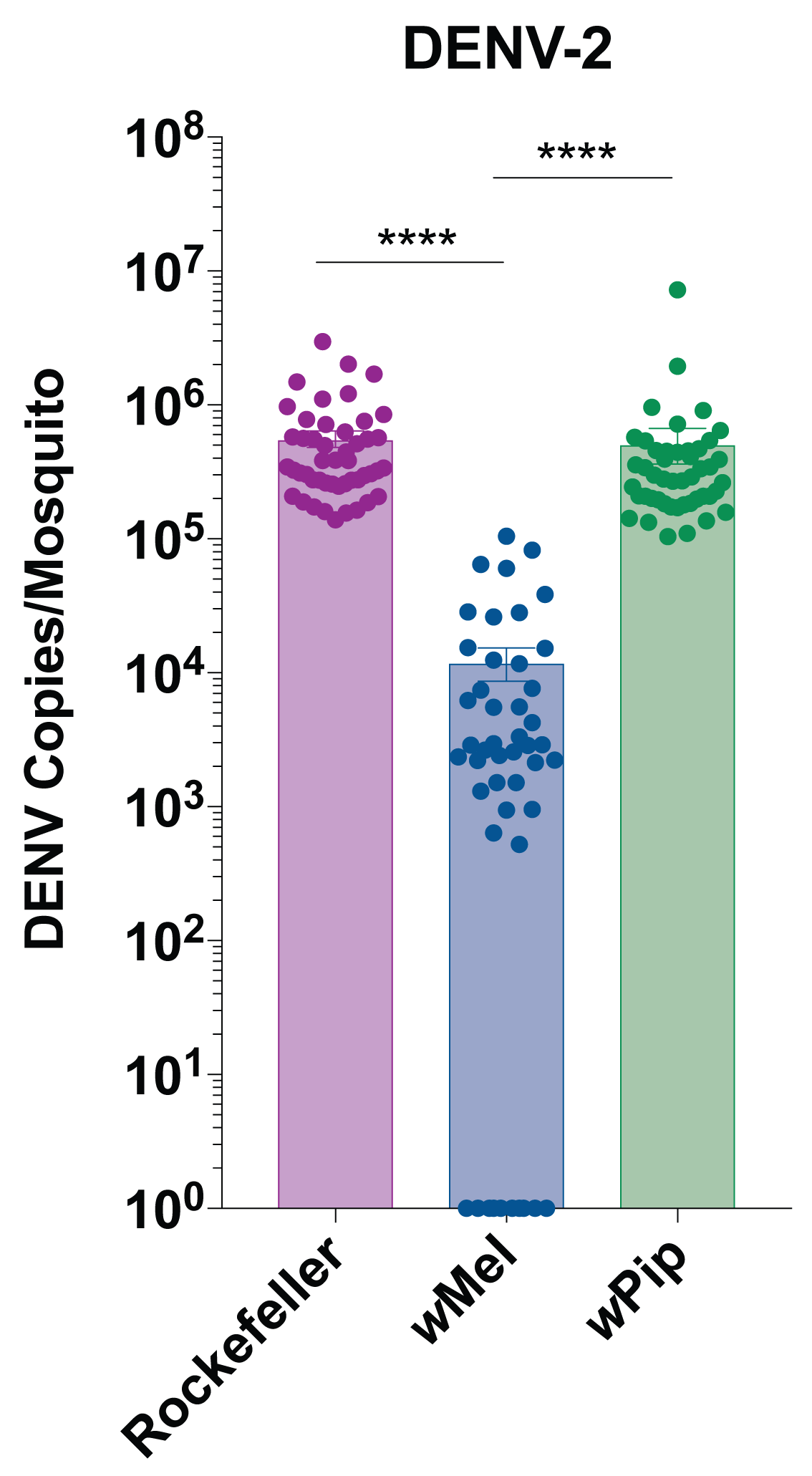

Supplement: S2 Fig — DENV-2 (isolated from a patient in Vietnam in 2010) [37] was injected into the thorax of 7-day old female mosquitoes at 2.4 x10^5^ TCID50/ml. RNA was extracted from whole mosquito bodies 7-days post infection and virus replication was quantified by qRT-PCR. Data are the mean number of DENV genome copies per mosquito ± SEM with individual data points overlaid. Asterisks indicate significance compared to Rockefeller (no Wolbachia control) (Kruskal-Wallis test with Dunn’s correction; ****p<0.0001). (TIF) [file ppat.1008410.s002.tif]
